# Data Resource Profile: Device-measured physical activity and sleep in the HUNT4 study, Norway

**DOI:** 10.1093/ije/dyag125

**Published:** 2026-08-10

**Authors:** Vegar Rangul, Atle Kongsvold, Saraswoti Lamsal Khanal, Mats Flaaten, Eivind Schjelderup Skarpsno, Kerstin Bach, Tom Ivar Lund Nilsen, Paul Jarle Mork

**Affiliations:** HUNT Research Centre, Department of Public Health and Nursing, Faculty of Medicine and Health Sciences, Norwegian University of Science and Technology (NTNU), Levanger, Norway; Levanger Hospital, Nord-Trøndelag Hospital Trust, Levanger, Norway; Department of Public Health and Nursing, Faculty and Medicine and Health Sciences, Norwegian University of Science and Technology (NTNU), Trondheim, Norway; HUNT Research Centre, Department of Public Health and Nursing, Faculty of Medicine and Health Sciences, Norwegian University of Science and Technology (NTNU), Levanger, Norway; Department of Public Health and Nursing, Faculty and Medicine and Health Sciences, Norwegian University of Science and Technology (NTNU), Trondheim, Norway; Department of Public Health and Nursing, Faculty and Medicine and Health Sciences, Norwegian University of Science and Technology (NTNU), Trondheim, Norway; Department of Computer Science, Norwegian University of Science and Technology (NTNU), Trondheim, Norway; Department of Public Health and Nursing, Faculty and Medicine and Health Sciences, Norwegian University of Science and Technology (NTNU), Trondheim, Norway; Clinic of Emergency Medicine and Prehospital Care, St. Olav’s Hospital, Trondheim University Hospital, Trondheim, Norway; Department of Public Health and Nursing, Faculty and Medicine and Health Sciences, Norwegian University of Science and Technology (NTNU), Trondheim, Norway

**Keywords:** HUNT study, cohort studies, longitudinal studies, accelerometry, 24-hour movement behaviours, physical activity, sleep, machine learning

Key Features

- The Trøndelag Health Study (HUNT), initiated in 1984–6 in mid-Norway, is a population-based study with four consecutive surveys among adults (HUNT1–4, ≥20 years) and adolescents (Young-HUNT1–4, 13–19 years) that comprises health data from ∼230 000 individuals.
- In HUNT4 and Young-HUNT4 (2017–19), 38 756 participants consented to wear two tri-axial accelerometers (on thigh and lower back) for ≤7 days and 32 644 individuals (27 329 adults; 5315 adolescents) provided at least 1 day with valid accelerometer measurements.
- Accelerometer data are processed by using validated machine-learning models to predict key activity types (walking, running, cycling), postures (lying, sitting, standing), and sleep duration. Algorithms and training datasets are publicly available for reproducibility.
- HUNT data can be linked to national health registries via a unique personal identification number, enabling longitudinal and intergenerational research on morbidity, mortality, prescriptions, and healthcare utilization.
- Data access is available through the HUNT Databank (https://www.ntnu.edu/hunt/research) upon ethics approval and collaboration with a Norwegian principal investigator. Documentation, variable lists, and protocols are provided to support transparent and secure data use.

## Data resource basics

Over six decades of epidemiological research have firmly established physical activity as a key factor in promoting lifelong health and preventing disease [1–4]. More recently, sedentary behaviour and insufficient sleep have been recognized as independent contributors to health outcomes [5, 6]. Large-scale population-based studies have traditionally relied on self-reports to assess physical activity, sedentary behaviour, and sleep duration. However, self-reports are prone to measurement error and bias [7–9], which may hamper further progress in the field, whereas device-based measurements capture free‑living movement accrued across the day, including unstructured and unplanned physical activity. To better understand the impact of the full 24-hour activity cycle on health and disease, future research should adopt state-of-the-art device-based measurement techniques [10–13]. The output of this research can support the development of targeted interventions and provide a foundation for evidence-based public health recommendations on the optimal balance between physical activity, sedentary behaviour, and sleep.

The Trøndelag Health Study (HUNT) is an ongoing population-based cohort study in Trøndelag county, mid-Norway. Since 1984–6, all residents in the study area have been invited to repeated health surveys, comprising four adult surveys (HUNT1–4, ≥20 years) and, since 1995–7, four adolescent surveys (Young-HUNT1–4, 13–19 years). Across all survey cycles, HUNT currently includes data from *∼*230 000 unique participants. Adults aged ≥20 years in the Nord‑Trøndelag region were invited to HUNT1 (1984–6, 77 202 participants, 89.4%), HUNT2 (1995–7, 65 228 participants, 69.5%), HUNT3 (2006–8, 50 800 participants, 54.1%), and HUNT4 (2017–19, 56 042 participants, 54.0%) [14, 15]. Additionally, all adolescents aged 13–19 years in the Nord-Trøndelag region have been included in four Young-HUNT surveys. Young-HUNT1 (1995–7, 8980 participants, 88.0%) was a school-based, cross-sectional survey inviting all children aged 13–19 years living in Nord-Trøndelag. Young-HUNT2 (2000–1, 2427 participants, 77.0%) was a follow‑up survey inviting previous Young‑HUNT1 participants who still resided in the region, rather than a new general invitation. Young-HUNT3 (2006–8, 8199 participants, 78.4%) [16] and Young-HUNT4 (2017–19, 8066 participants, 76.0%) were new cross‑sectional surveys in which all adolescents aged 13–19 years living in the study area were invited. The HUNT study includes data on a broad range of health-related topics collected via questionnaires, interviews, clinical examinations, laboratory tests, and biological sampling. Although this Data Resource Profile focuses on HUNT4, including Young-HUNT4, we provide the full HUNT and Young-HUNT context because the shared infrastructure, harmonized protocols, and long-term follow-up across survey waves are essential for understanding the design and research potential of HUNT4. Detailed information about the data collected in HUNT and Young-HUNT can be found in previous cohort profile publications [15, 17] and on the HUNT study website (https://www.ntnu.edu/hunt/data/que).

The HUNT study is funded by the Norwegian Ministry of Health, the Norwegian University of Science and Technology (NTNU), Trøndelag County Council, and the Central Norway Regional Health Authority. The next HUNT survey (HUNT5/Young-HUNT5) is planned for 2028–30. The HUNT study operates under General Data Protection Regulation (GDPR)‑compliant Standard Operating Procedures with ethics approval and informed consent. The unique personal identification number assigned to all Norwegian residents enables linkages with a broad range of national registries, including those covering morbidity, mortality, prescriptions, and healthcare utilization. The cohort is dynamic and participant numbers may change over time due to ongoing data-quality checks, updated linkage information, and withdrawals of consent. In accordance with GDPR and Norwegian regulations, participants may request deletion of their data at any time and such requests are implemented promptly. As a result, descriptive statistics and total sample counts may vary slightly between data releases.

In HUNT4 and Young‑HUNT4, participants were invited to wear dual-site accelerometers for 7 days to assess physical activity, postures, and sleep. In the present profile, we describe the accelerometer ‘sub-cohort’ from the fourth survey wave, HUNT4 (2017–19), which is cross‑sectional in design and nested within the ongoing longitudinal HUNT cohort. These data are available via the HUNT Databank upon ethics approval and collaboration with a Norwegian principal investigator. The protocol, processing pipeline, and characteristics of the participants wearing accelerometers are detailed below.

## Data collected

Data on socio-economic status, health conditions, symptoms, and health-related behaviours were collected via questionnaires in all HUNT surveys. All eligible adults and adolescents residing in the study area were invited to participate in each survey, ensuring a population-based sample. Questionnaire content, clinical-examination protocols, and biological sampling procedures have evolved across surveys, reflecting advancements in methodology and shifting research priorities. In addition to the core data collection, a range of ancillary studies have been conducted, such as audiometric testing, brain magnetic resonance imaging, echocardiography, and celiac disease screening. Data-harmonization procedures are implemented to ensure comparability across surveys and alignment with international cohorts. Prior to delivery to researchers, HUNT datasets are pseudo anonymized, with safeguards applied to quasi-identifiers to minimize re-identification risk. Data are delivered via approved secure transfer methods and storage must comply with institutional safeguards, ethics approval, and GDPR-compliant procedures.

### Device-based measurements of physical activity, postures, and sleep

Participants in both HUNT4 and Young-HUNT4 were invited to wear two tri-axial AX3 accelerometers (Axivity, Ltd, Newcastle, UK) for a period of 7 consecutive days. The AX3 is small (23 × 32.5 × 7.6 mm; 11 g) and waterproof, and has a 512-MB internal flash drive for data storage. The accelerometers were placed centrally on the right thigh (∼10 cm above the upper border of the patella) and centrally on the third lumbar segment (L3) on the lower back. Trained personnel attached the sensors by (i) applying an adhesive film (5 × 7 cm, Opsite Flexifix; Smith & Nephew, Watford, UK) on the skin, (ii) securing the sensor on top of the film by using double-sided tape (3M, St. Paul, MN, USA), and (iii) covering the sensor with a second layer of adhesive film (10 × 8 cm). The OmGui software (version 1.0.0.43; Open Movement, Newcastle, UK) was used to configure, initialize, and download the data. Data were sampled at 50 Hz with ±8G bandwidth, stored on the internal flash drive, and thereafter downloaded for visualization and further analysis.

**Figure 1:**
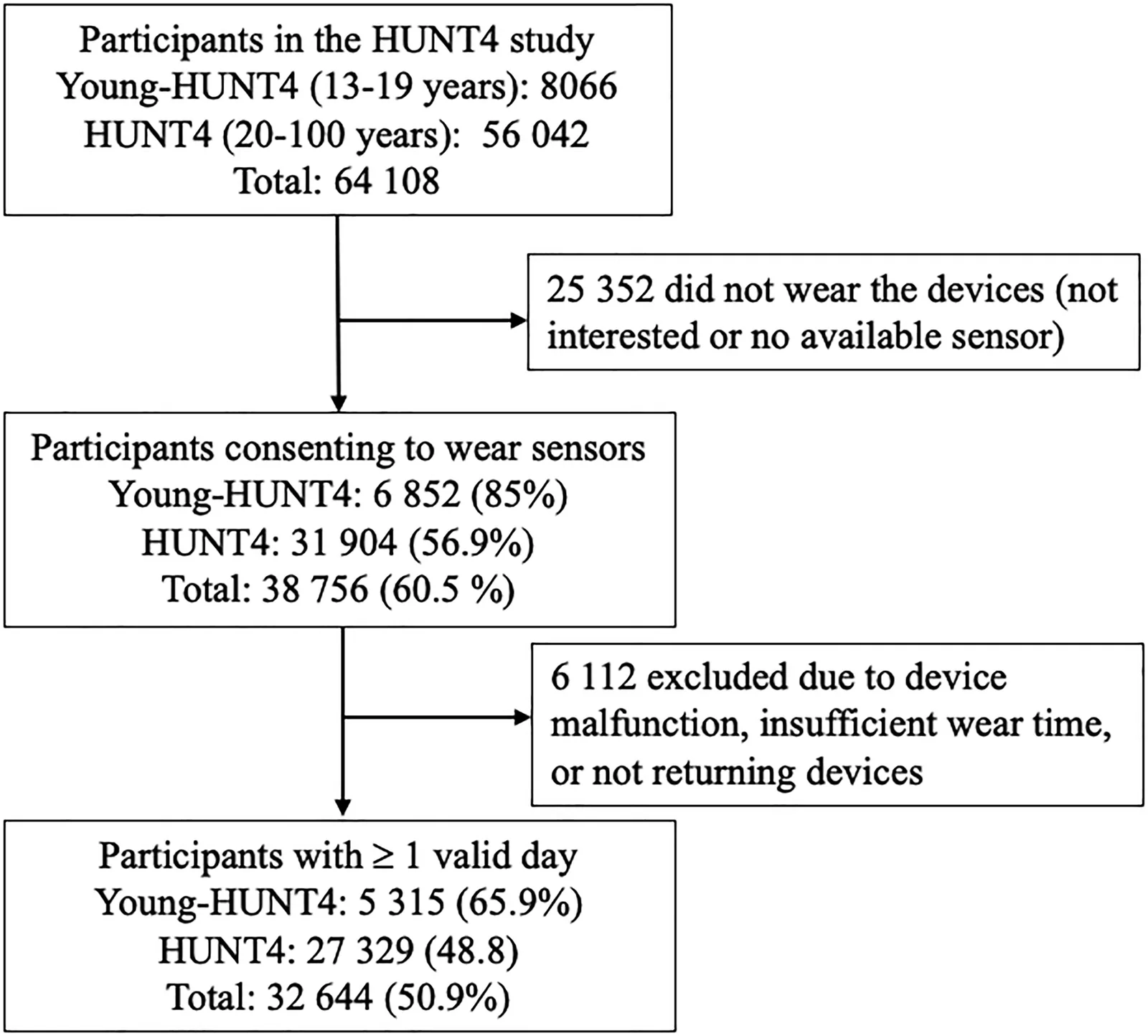
Flow chart of the accelerometer data collection in HUNT4 and Young-HUNT4 (2017–19).

Of the 56 042 (54%) adults and 8066 (76.0%) adolescents who participated in HUNT4 and (Young-HUNT4), a total of 38 756 consented to wear accelerometers (Fig. 1). Of these, 32 644 participants had a ≥1 valid day of accelerometer data (48.8%; 27 329 adults and 65.9%; 5315 adolescents) (Fig. 1 and Table 1). Machine-learning models were developed to predict the daily pattern of key physical-activity types [slow walking (≤4 km/h), moderate walking (4.1–5.4 km/h), brisk walking (≥5.5 km/h), running, and cycling], postures (lying, sitting, standing), and sleep, with high overall accuracy ranging from 84% to 96% [18–21]. Moderate-to-vigorous physical activity (MVPA) was defined as time classified by the machine-learning models as moderate walking, brisk walking, running, or cycling. The total walking time, in contrast, includes all walking speeds [slow, (≤4 km/h), moderate, and brisk walking]. Consequently, a large proportion of total walking reflects slow, light-intensity walking that does not contribute to the MVPA variable based on these absolute speed thresholds. To process the accelerometer data, the two downloaded files from each participant were synchronized and then segmented into 5-s windows (250 samples), before 161 different features were computed for each window. These features were fed into an eXtreme Gradient Boosting machine-learning model trained to predict the key physical activities and postures listed above [19, 21]. Separate machine-learning models were trained to predict sleep duration [18] and no-wear time [22]. If no-wear time was predicted for ≥1 hour, then the entire 24 hours for that day were excluded from the data material. The days on which were mounted and removed were also excluded (i.e. the first and last day of measurements). Consequently, only days with complete 24-hour accelerometer recordings (from midnight to midnight) are included in the data material. The training data and parameter setting used to configure the machine-learning classifiers are publicly available (https://github.com/ntnu-ai-lab/harth-ml-experiments).

**Table 1:** Characteristics of participants in HUNT4 and Young-HUNT4 with ≥1 valid day of accelerometer recording. Values are numbers (%) unless otherwise stated.

Characteristics	Adolescents aged 13–19 years		Adults aged ≥20 years	
	Females	Males	Females	Males
	(*n *= 3005)	(*n *= 2310)	(*n *= 15 534)	(*n *= 11 795)
Age, (years) [mean (SD)]	16.1 (1.8)	16.1 (1.8)	53.9 (17.8)	54.8 (17.3)
Days of valid accelerometry, median (interquartile range)	6 (0)	6 (1)	6 (0)	6 (0)
≤2	187 (6.2)	307 (13.3)	816 (5.3)	510 (4.3)
3	102 (3.4)	160 (6.9)	379 (2.4)	268 (2.3)
4	178 (5.9)	162 (7.0)	563 (3.6)	416 (3.5)
5	322 (10.7)	307 (13.3)	1777 (11.4)	1259 (10.7)
6	1910 (63.6)	1164 (52.4)	10 991 (70.8)	8340 (72.7)
7	306 (10.2)	210 (9.1)	1008 (6.5)	1102 (8.5)
Educational attainment (adults)				
Primary school			1570 (10.2)	961 (8.2)
Upper secondary school			6645 (43.0)	6296 (53.6)
University or college			7241 (46.9)	4487 (38.2)
Educational aspirations (adolescents)				
No plans/other education	1012 (33.9)	814 (35.7)		
Upper secondary school	376 (12.6)	624 (27.3)		
University or college	1597 (53.5)	844 (37.0)		
Yearly household income (EUR)				
<38 000	272 (9.2)	148 (6.5)	4796 (31.8)	2911 (25.0)
38 000–85 000	2224 (74.8)	1584 (69.2)	7571 (50.3)	6329 (54.4)
>85 000	476 (16.0)	556 (24.3)	2700 (17.9)	2386 (20.5)
Smoking status				
Not smoking	2906 (96.5)	2223 (95.8)	14 038 (90.4)	10 928 (92.7)
Current smoker	104 (3.5)	97 (4.2)	1497 (9.6)	866 (7.3)
Alcohol (units/week)				
<1	156 (12.3)	110 (13.5)	2851 (20.6)	1231 (11.1)
1–4	757 (59.8)	358 (44.0)	9413 (68.0)	6712 (60.5)
≥5	353 (27.9)	345 (42.4)	1574 (11.4)	3160 (28.5)
Self-rated health				
Poor/not good	455 (15.2)	212 (9.2)	3468 (22.7)	2158 (18.5)
Good	1822 (61.0)	1339 (58.2)	8991 (58.8)	7264 (62.3)
Very good	708 (23.7)	750 (32.6)	2836 (18.5)	2246 (19.3)
Body mass index^a^ (kg/m^2^) [mean (SD)]	22.5 (4.2)	21.6 (4.0)	26.7 (4.8)	27.4 (4.1)
Underweight (<18.5)	166 (5.6)	142 (6.1)	211 (1.4)	35 (0.3)
Normal (18.5–24.9)	2088 (69.9)	1696 (73.5)	6024 (39.3)	3229 (27.6)
Overweight (25.0–29.9)	541 (18.1)	337 (14.6)	5722 (37.3)	5780 (49.5)
Obese (≥30.0)	193 (6.5)	134 (5.8)	3380 (22.0)	2639 (22.6)

a Age and gender adjustments for adolescents by International Obesity Task Force (IOTF) (Cole *et al.*, 2000).

Table 1 presents characteristics of the participants in HUNT4 and Young-HUNT4 who had ≥1 day of valid accelerometer recording. The mean age was 16.1 (SD 1.8) years in both male and female adolescents, while the mean age was 53.9 (SD 17.7) and 54.8 (SD 17.3) years in adult females and males, respectively. The median number of days with valid accelerometer recordings was 6 days (IQR 0) for adolescent females, 6 days (IQR 1) for adolescent males, and 6 days (IQR 0) for both adult females and adult males. The proportion of participants with ≥3 valid days of recording was high across all groups: 93.8% in female adolescents, 86.7% in male adolescents, 94.7% in adult females, and 95.7% in adult males. Participant counts reflect the current curated dataset; minor changes may occur because of consent withdrawals or ongoing data-quality procedures.

Among adolescents, a higher proportion of females than males reported university or college aspirations (53.5% vs. 37.0%). Among adults, higher educational attainment was more common in females (46.9%) than in males (38.2%). Household-income distributions were skewed toward the middle-income group (Eurostat classified income group) in all age groups, with adult males slightly more likely to be in the highest-income category. Proportions of current smokers were low in both adolescents (3.5% in females, 4.2% in males) and adults (9.6% in females, 7.3% in males). More males than females reported consuming ≥5 alcohol units per week (adults: 28.5% vs. 11.4%; adolescents: 42.4% vs. 27.9%). Proportions rating their health as good or very good were high in both adolescents (84.7% females, 90.8% males) and adults (77.3% females, 81.6% males). Body mass index distributions [International Obesity Task Force (IOTF) categories] [23] indicated that most adolescents had normal weight (69.9% females, 73.5% males), whereas, among adults, overweight (37.3% females, 49.5% males) and obesity (22.0% females, 22.6% males) were more common.

Table 2 presents the median values of the participants’ average daily time spent in physical activity, postures, and sleep among the 32 644 participants with ≥3 valid days of accelerometer measurements in HUNT4 and Young-HUNT4. The median durations varied across age, sex, and health characteristics such as self-rated health, smoking status, body mass index, and metabolic health. Overall, males tended to accumulate more time performing MVPA (32.7 vs. 28.3 min/day), sitting (500 vs. 472 min/day), and lying down (147 vs. 125 min/day) than females, whereas females tended to spend more time standing (278 vs. 243 min/day) and sleeping (428 vs. 410). MVPA was highest in adolescents (41.4 min/day) and declined with age, particularly after age 60 years. Sitting time increased with age, while sleep duration was longest among adolescents and older adults (∼7.2 hours/day). Participants with higher education or income achieved more MVPA but also more sitting time than those with lower socio-economic status. Furthermore, non-smokers and those reporting good or very good health undertook more MVPA and had longer sleep, whereas overweight or obese participants engaged in less MVPA and more sitting.

**Table 2:** Daily duration of physical activity, posture, and sleep by demographic and health characteristics among participants with ≥1 day with valid accelerometer data. Values are median and interquartile range (IQR) unless stated otherwise.

Characteristics	*N* (%)	MVPA^a^ (min/day)	Walking (min/day)	Standing (min/day)	Sitting (min/day)	Lying down^b^ (min/day)	Sleep duration (min/day)
Sex							
Female	18 539 (56.8)	28.3 (16.1–43.2)	93.8 (71.0–117.9)	278 (225–335)	472 (403–547)	125 (80.9–196)	428 (399–453)
Male	14 105 (43.2)	32.7 (19.6–49.4)	94.8 (71.3–121.1)	243 (190–302)	500 (416–583)	147 (94–224)	410 (380–437)
Age group (years)							
13–19	5315 (16.3)	41.4 (28.8–57.4)	82.1 (62.6–105.6)	212 (172–254)	441 (374–508)	228 (168–311)	434 (407–457)
20–29	2878 (8.8)	32.0 (21.1–45.2)	87.9 (66.3–110.6)	264 (212–322)	426 (359–499)	198 (140–277)	422 (390–450)
30–39	3449 (10.6)	31.4 (20.6–44.3)	100.7 (79.2–124.2)	307 (253–358)	431 (366–501)	150 (102–216)	414 (387–441)
40–49	4584 (14.0)	32.7 (21.5–47.7)	103.0 (81.8–127.6)	298 (246–354)	467 (400–536)	120 (82–179)	412 (385–438)
50–59	5451 (16.7)	31.8 (19.9–47.4)	104.0 (81.7–128.5)	282 (231–338)	502 (428–573)	109 (72–164)	410 (382–435)
60–69	5566 (17.1)	26.8 (15.1–42.1)	100.1 (76.6–124.8)	263 (213–319)	522 (448–594)	103 (68–153)	419 (390–447)
70–79	4123 (12.6)	17.4 (7.8–32.2)	85.3 (61.7–110.4)	245 (197–302)	548 (472–621)	103 (67–152)	429 (397–456)
80–89	1123 (3.4)	6.5 (2.5–15.5)	60.6 (41.8–83.5)	232 (177–289)	575 (493–647)	115 (69–173)	432 (402–460)
≥90	155 (0.5)	1.9 (1.1–5.3)	34.9 (17.4–52.8)	214 (142–286)	594 (513–659)	119 (76–186)	439 (410–469)
Educational attainment (adults)							
Other education/primary school	2531 (9.3)	15.1 (6.2–29.4)	80.9 (56.2–105.7)	254 (200–310)	532 (447–612)	115 (74–176)	427 (393–457)
Upper secondary school	12 942 (47.6)	27.0 (15.4–42.2)	97.6 (72.7–123.6)	275 (219–335)	488 (409–572)	124 (81–190)	415 (385–444)
University or college	11 728 (43.1)	31.4 (19.5–46.1)	99.1 (77.5–122.0)	279 (227–334)	491 (417–563)	117 (78–177)	418 (391–443)
Educational aspirations (adolescents)							
No plans/other education	1826 (34.7)	42.7 (29.8–58.7)	82.8 (62.9–105.8)	211 (171–252)	437 (369–502)	233 (170–322)	435 (407–456)
Upper secondary school	1000 (19.0)	41.2 (29.4–58.5)	82.2 (60.6–107.0)	205 (166–247)	428 (357–500)	243 (178–336)	431 (402–456)
University or college	2441 (46.4)	40.5 (28.1–56.0)	81.4 (62.8–104.7)	216 (176–257)	448 (386–515)	221 (163–295)	434 (409–459)
Yearly household income (EUR)							
<38 000	8127 (25.4)	21.5 (9.8–37.7)	84.5 (60.1–111.0)	254 (200–315)	503 (414–590)	132 (83–208)	424 (391–453)
38 000–85 000	17 708 (55.4)	31.7 (19.7–47.0)	96.9 (74.2–121.5)	265 (211–325)	475 (404–553)	136 (87–211)	420 (361–447)
>85 000	6118 (19.2)	35.6 (23.9–51.1)	100.1 (78.3–123.1)	269 (284–326)	484 (412–555)	127 (84–197)	420 (389–441)
Smoking status							
Not smoking	25 811 (91.0)	29.1 (16.9–44.5)	96.8 (73.4–121.7)	273 (219–331)	490 (413–568)	122 (80–187)	419 (389–446)
Current smoker	2564 (9.0)	21.5 (11.7–35.0)	88.4 (64.4–113.5)	262 (203–326)	501 (419–582)	140 (88–217)	411 (381–441)
Alcohol (units/week)							
<1	4348 (16.1)	23.9 (11.7–38.6)	89.8 (63.8–116.4)	274 (216–337)	482 (404–568)	130 (84–202)	419 (389–449)
1–4	17 240 (63.8)	29.9 (18.0–44.9)	97.4 (74.4–121.9)	275 (221–333)	488 (414–564)	122 (80–187)	419 (390–445)
≥5	5432 (20.1)	31.7 (19.6–47.3)	96.4 (73.6–119.8)	254 (204–309)	499 (423–576)	136 (88–208)	414 (386–442)
Self-rated health							
Poor/not good	6293 (19.5)	19.2 (9.1–32.6)	79.4 (57.0–105.8)	249 (196–307)	508 (424–592)	139 (87–216)	423 (391–453)
Good	19 416 (60.2)	30.4 (18.6–45.2)	95.7 (73.3–120.5)	265 (212–325)	484 (409–563)	131 (85–204)	420 (391–447)
Very good	6540 (20.3)	39.9 (27.2–56.2)	102.6 (80.8–126.5)	271 (218–331)	461 (393–534)	139 (88–214)	418 (391–444)
Body mass index^c^ ( kg/m^2^)							
Underweight (<18.5)	556 (1.7)	36.0 (20.9–52.6)	84.3 (62.1–113.0)	239 (188–298)	430 (355–503)	206 (143–307)	435 (403–459)
Normal (18.5–24.9)	13 056 (40.3)	36.0 (22.7–52.0)	98.0 (74.1–122.5)	263 (209–324)	455 (387–526)	154 (97–234)	425 (396–450)
Overweight (25–30)	12 399 (38.3)	28.9 (16.9–43.8)	96.8 (74.6–121.0)	266 (215–325)	497 (420–571)	124 (82–189)	418 (389–445)
Obese (>30)	6361 (19.7)	22.0 (11.4–35.0)	82.7 (60.8–107.3)	258 (203–319)	528 (444–616)	117 (73–185)	413 (382–444)

a MVPA was defined as cycling, running, moderate walking (4.1–5.4 km/h), and brisk walking (>5.4 km/h), assessed among participants with ≥3 valid days by using absolute speed‑based thresholds for walking.

b Sleep not included.

c Age and gender adjustments for adolescents by IOTF (Cole *et al.*, 2000).

## Data resource use

The dataset can be used as an outcome to examine determinants and correlates of physical activity, as a resource for validating survey measures, and as an exposure to prospectively examine associations with health outcomes [24, 25]. For example, intergenerational and genetic data from HUNT have been used to examine the association between parental physical activity and that of their adult offspring [26]. The most active offspring are those with high genetic liability for physical activity and physically active parents, particularly when both parents have a history of being active. Physical activity in adulthood is also strongly and dose-dependently associated with participation in sports and exercise during adolescence [24]. The dataset has been used further to examine the way in which age and season influence MVPA and total physical activity [27]. As expected, MVPA declines with age and varies substantially by season, most notable among adolescents and younger adults. Across all age groups, moderate-intensity and brisk walking contribute the most to MVPA, while slow walking dominates total physical activity, especially among older adults.

A separate line of research has focused on the validation of survey measures. One study evaluated whether statistical correction models could improve the accuracy of self-reported sitting time by using device-measured sitting as the reference standard [28]. The results indicated only marginal improvement, emphasizing the need for objective monitoring or improved reporting methods for measuring sitting time.

Using a prospective study design, our research has shown that the volume and intensity of device-measured daily walking are inversely and dose-dependently associated with risk of chronic low back pain [29]. In reproductive health research, pre-pregnancy device-measured physical activity, linked to the Medical Birth Registry of Norway, was associated with lower odds of gestational diabetes and a composite adverse pregnancy outcome among 700 women [30].

Table 3 presents the proportions of participants wearing accelerometers in HUNT4 and Young-HUNT4 according to demographic and health-related factors, along with adjusted predicted probabilities of wearing sensors. Overall, a higher proportion of females (55.0%) than males (50.1%) wore accelerometers. Participation peaked in adolescence (females 76.5%, males 63.5%) but both the youngest and oldest adult participants showed low participation (≥90 years; females 27.5%, males 30.3%). Participants with university education or higher income had greater predicted probabilities of wearing sensors than those with lower education or income. Individuals reporting good or very good self-rated health and those with a normal body mass index were more likely to wear sensors than those reporting poor health or who were obese. Smokers were less likely to wear sensors than non-smokers, while no clear difference was observed between levels of alcohol consumption. Furthermore, there was a clear gradient by self-reported physical activity, with higher predicted probabilities of wearing accelerometers among participants reporting moderate (women 0.56, men 0.51) and high physical activity (women 0.59, men 0.53) compared with those reporting low activity (women 0.45, men 0.44). These results indicate modest but systematic selection in wearing an accelerometer. This should be taken into account in future analyses (e.g. through descriptive comparison, sensitivity checks, or other feasibility-appropriate approaches) when generalizing accelerometer-based findings to the broader HUNT4 and Young-HUNT4 populations.

**Table 3:** Descriptive characteristics and predicted probability of wearing sensors (‘Yes’) based on characteristics observed among participants who did not wear sensors (‘No’). Values are number (%). Probabilities were estimated by using logistic regression with predictive margins.

Characteristics	**Females**			**Males**		
	No^a^	Yes^b^	Predicted probability^c^ (95% CI)	No^a^	Yes^b^	Predicted probability^c^ (95% CI)
	*n *= 15 367	*n *= 18 539		*n *= 15 224	*n *= 14 105	
**Age group (years)**						
13–19	924 (23.5)	3010 (76.5)	0.77 (0.75–0.78)	1333 (36.5)	2320 (63.5)	0.64 (0.62–0.65)
20–29	1677 (48.8)	1759 (51.2)	0.51 (0.50–0.53)	1491 (57.1)	1119 (42.9)	0.42 (0.38–0.47)
30–39	1703 (45.2)	2067 (54.8)	0.55 (0.53–0.56)	1365 (49.7)	1382 (50.3)	0.57 (0.52–0.64)
40–49	2203 (44.5)	2752 (55.5)	0.56 (0.54–0.57)	1930 (51.3)	1832 (48.7)	0.54 (0.49–0.59)
50–59	2569 (44.9)	3153 (55.1)	0.55 (0.54–0.56)	2479 (51.9)	2298 (48.1)	0.53 (0.48–0.57)
60–69	2771 (48.6)	2935 (51.4)	0.51 (0.50–0.53)	2720 (50.8)	2631 (49.2)	0.55 (0.50–0.60)
70–79	2153 (49.9)	2164 (50.1)	0.50 (0.49–0.52)	1981 (50.3)	1959 (49.7)	0.56 (0.51–0.62)
80–89	1106 (64.6)	606 (35.4)	0.35 (0.33–0.38)	796 (60.6)	517 (39.4)	0.37 (0.32–0.42)
≥90	261 (72.5)	99 (27.5)	0.28 (0.23–0.32)	129 (69.7)	56 (30.3)	0.24 (0.18–0.34)
Educational attainment (adults)						
Other education/primary school	2366 (60.1)	1570 (39.9)	0.40 (0.38–0.41)	1532 (61.5)	961 (38.5)	0.39 (0.37–0.40)
Upper secondary school	6551 (49.6)	6646 (50.4)	0.50 (0.50–0.51)	7287 (53.6)	6297 (46.4)	0.46 (0.48–0.47)
University or college	5316 (42.3)	7241 (57.7)	0.58 (0.57–0.59)	3954 (46.8)	4487 (53.2)	0.53 (0.52–0.54)
Educational aspirations (adolescents)						
No plans/other education	303 (23.0)	1012 (77.0)	0.77 (0.75–0.79)	493 (37.6)	817 (62.4)	0.62 (0.60–0.65)
Upper secondary school	96 (20.3)	377 (79.7)	0.80 (0.76–0.83)	339 (35.1)	626 (64.9)	0.65 (0.62–0.68)
University or college	518 (24.5)	1598 (75.5)	0.76 (0.74–0.77)	489 (36.7)	845 (63.3)	0.63 (0.61–0.66)
Yearly household income (EUR)						
<38 000	5546 (38.1)	5068 (28.1)	0.50 (0.49–0.51)	3888 (28.0)	3059 (22.0)	0.45 (0.44–0.47)
38 000–85 000	6943 (47.7)	9795 (54.3)	0.57 (0.57–0.58)	7331 (52.8)	7913 (56.9)	0.51 (0.51–0.52)
>85 000	2057 (14.1)	3176 (17.6)	0.61 (0.59–0.62)	2662 (19.2)	2942 (21.1)	0.52 (0.51–0.53)
Smoking status						
Not smoking	13 603 (44.2)	17 167 (55.8)	0.55 (0.55–0.56)	12 798 (49.0)	13 322 (0.51)	0.51 (0.50–0.51)
Current smoker	1764 (52.4)	1601 (47.6)	0.49 (0.48–0.51)	1426 (59.7)	963 (0.40.3)	0.41 (0.39–0.43)
Alcohol (units/week)						
<1	3330 (52.5)	3007 (47.5)	0.48 (0.47–0.49)	1613 (54.6)	1341 (45.4)	0.45 (0.44–0.47)
1–4	8037 (44.1)	10 170 (55.9)	0.56 (0.55–0.57)	7201 (50.5)	7070 (49.5)	0.50 (0.49–0.50)
≥5	1404 (42.1)	1927 (57.8)	0.56 (0.55–0.58)	3533 (50.2)	3505 (49.8)	0.50 (0.48–0.51)
Self-rated health						
Poor/not good	4499 (53.4)	3923 (46.6)	0.48 (0.47–0.49)	3266 (57.9)	2370 (42.1)	0.43 (0.42–0.44)
Good	8375 (43.6)	10 813 (56.4)	0.56 (0.56–0.57)	8417 (49.5)	8603 (50.5)	0.51 (0.50–0.51)
Very good	2201 (38.3)	3544 (61.7)	0.61 (0.59–0.62)	2361 (44.1)	2996 (55.9)	0.55 (0.53–0.56)
Body mass index (kg/m^2^)						
Underweight (<18.5)	281 (1.9)	379 (2.1)	0.52 (0.49–0.56)	128 (0.9)	177 (1.3)	0.50 (0.44–0.56)
Normal (18.5–24.9)	5653 (38.1)	8123 (44.3)	0.57 (0.56–0.58)	4101 (29.5)	4933 (35.2)	0.52 (0.51–0.53)
Overweight (25–30)	5116 (34.5)	6270 (34.2)	0.56 (0.55–0.57)	6254 (45.0)	6129 (43.7)	0.51 (0.50–0.51)
Obese (>30)	3783 (25.5)	3580 (19.5)	0.51 (0.49–0.52)	3422 (24.6)	2781 (19.8)	0.46 (0.45–0.47)
Self-reported physical activity (min/week)						
Low (<60)	2779 (54.8)	2294 (45.2)	0.45 (0.44–0.47)	3174 (56.2)	2467 (43.7)	0.44 (0.43–0.46)
Moderate (60–150)	8884 (44.6)	11 023 (55.4)	0.56 (0.55–0.56)	7785 (49.5)	7941 (50.5)	0.51 (0.50–0.52)
High (>150)	3297 (40.1)	4928 (59.9)	0.59 (0.58–0.60)	2941 (45.7)	3496 (54.3)	0.53 (0.52–0.54)

a Percentage within the total number of HUNT4 participants who did not wear movement sensors (No).

b Percentage within the total number of HUNT4 participants who wore movement sensors (Yes).

c Predicted probabilities of wearing sensors (Yes) estimated by using logistic regression with predictive margins (Stata margins command).

## Strengths and weaknesses

Strengths of this data resource include the use of dual-site accelerometers, which enables detailed and accurate classification of daily movement behaviours and sleep across the 24‑hour cycle. The large, population-based sample covering adolescents to older adults provides substantial breadth for subgroup analyses and enhances the representativeness of the data. Wear-time compliance was high, and both the sensor protocol and machine-learning algorithms have been validated within the HUNT population, supporting the reliability of the derived measures. The integration of accelerometer data into an established longitudinal cohort with linkage to national health registries strengthens opportunities for long‑term, intergenerational, and clinically relevant follow‑up. At the same time, selection bias should be considered, as individuals who agreed to wear accelerometers may differ systematically from non-participants in socio-economic and health characteristics. This may limit external validity and generalizability of the results, and should be addressed in analyses. The 7‑day measurement window may not fully capture habitual activity patterns, particularly given the known seasonal variation in movement behaviours [27]. As with all model-based classifications, some degree of misclassification is expected despite the good performance metrics. In particular, our MVPA variable is based on absolute, speed-defined thresholds for walking and does not fully capture the relative intensity of activity in older or less fit participants; thus, slow or moderate walking on an absolute scale may represent moderate intensity for these individuals, implying that MVPA is likely underestimated in these groups. Furthermore, activities that are not well characterized by the walking, running, or cycling patterns used for training (e.g. gardening, resistance training, or swimming) may be recorded as movement but not classified as MVPA. These limitations should be considered when analysing and interpreting results from the accelerometer data.

## Data resource access

All the data are stored in the HUNT Databank, with variable information available in Norwegian and English on the HUNT Databank website (https://hunt-db.medisin.ntnu.no/hunt-db/variablelist). Additional details on the data-collection procedures can be found in previously published cohort profile updates [15, 17].

Any research group with a principal investigator affiliated with a Norwegian research institution can apply for access to the HUNT data. This means that researchers outside Norway may access the data through collaboration with a Norwegian partner. All projects require approval from the Regional Committee for Medical and Health Research Ethics (REC) in Norway.

Some data may be subject to time-limited exclusivity for research teams that funded and conducted specific data collections. Information about biological material and analytical services is available from the HUNT Biobank (https://www.ntnu.edu/hunt/hunt-biobank). Linkages between HUNT data and external registries must be requested separately for each project and require approval from REC and the relevant registry owners. Detailed information about the application procedures and access conditions are provided on the HUNT website (https://www.ntnu.edu/web/hunt/data). Questions may be directed to the corresponding author: vegar.rangul@ntnu.no.

## Ethics approval

This study was approved by REC (reference no.: HUNT4 17/00426-7/GRA). HUNT4 was not processed and approved by REC as a normal project, as it was assessed as being outside REC’s mandate. Since 2019, population-based data and sample collections have been regulated by the National Regulations on Population-Based Health Surveys, Norway.

## Data Availability

See ‘Data resource access’, above.
